# Prediction and monitoring of accrual and rate of underrepresented biomedical research group using bayesian methods

**DOI:** 10.1186/s12874-026-02822-3

**Published:** 2026-03-19

**Authors:** Kaustubh S. Nimkar, Byron J. Gajewski, Dinesh Pal Mudaranthakam, Jeffery A. Thompson, Miranda E. Handke, Robert N. Montgomery, Akinlolu O. Ojo

**Affiliations:** 1 Department of Biostatistics & Data Science, University of Kansas Medical Center, Mail Stop 1026, 3901 Rainbow Blvd, Kansas City, KS 66160 USA; 2 Department of Internal Medicine, University of Kansas Medical Center, Kansas City, KS 66160 USA

**Keywords:** Clinical Trials, Posterior Predictive Distribution, Sample Size, Prior, Interim

## Abstract

**Background:**

There has been a recent push for biomedical research to incorporate more demographically, ethnically, and medically diverse cohorts – individuals whom the NIH designates as “underrepresented in biomedical research” (UBR). In clinical trials, researchers often set out to achieve target rates of UBR, yet there are no methods used to help achieve these targets. Prediction and monitoring of the rate of UBR (rUBR) – the proportion of designated UBR participants - is essential to ensure these targets are met.

**Methods:**

We propose a monitoring tool that can simultaneously predict overall accrual and rUBR. The rUBR predictive algorithm extends the Bayesian overall accrual model by adding a Bayesian beta-binomial model.

**Results:**

We apply our method to two real-world completed clinical trial datasets: ADORE (An Assessment of DHA On Reducing Early preterm birth) and Quit2Live - a clinical trial to examine disparities in quitting between African American and White adult smokers. By doing this, we show the usefulness of this method at various time points in these trials and demonstrate that it can be used to monitor overall accrual and the rUBR for future research, including the All of Us Research Program.

**Conclusion:**

The simultaneous prediction of both overall accrual and rUBR gained by this method adds a novel probabilistic method to allow researchers to improve the monitoring process of their trial.

**Supplementary Information:**

The online version contains supplementary material available at 10.1186/s12874-026-02822-3.

## Background

A clinical trial must accrue a sample population of sufficient size to satisfy its needs, and it must do so within a specific timeframe; participant accrual is an essential component of this process. However, successfully achieving target sample sizes can be difficult. More than 80% of trials do not meet their enrollment accrual targets [1, 2]. When accrual targets are not met by the end of enrollment, there are significant financial costs due to extended timelines and low enrollment [3] along with a substantial loss in statistical power, which raises concerns about the trials’ feasibility and rigor. For these reasons, a clinical trial’s success may depend on accurate prediction of trial accrual.

By predicting and monitoring accrual throughout a trial, researchers can determine whether the target accrual goal will be met by the end of the trial and potentially enact alternative recruitment procedures to remedy issues that arise. Several methods have been proposed to predict participant accrual. One is to consider participant recruitment as a Poisson process: Senn [4] developed an accrual model that follows a Poisson process and assumes equal recruitment rates across multiple centers. Anisimov and Fedorov [5] proposed using Poisson models across multi-center studies. Their model of participant accrual follows a Poisson-Gamma distribution, where the rate parameters vary across centers. Previous research conducted by Abbas [6] proposed a method of using Markov Chain Monte Carlo for simulation for continuous and discrete recruitment times in clinical trials.

Here, we expand on a prediction method [7] which proposes an exponential waiting time model for participant accrual. Their Bayesian model differs from other methods as it allows prior information to be incorporated into the model through an inverse gamma prior distribution of the exponential parameter. We use their model and extend their work by incorporating accrual prediction of demographic, ethnic, and rural groups simultaneously. Recently, in biomedical research, there has been a push to engage with cohorts that are demographically, ethnically, and medically diverse [8] and to enroll a group of participants that better reflects the U.S. population [9]. To address this issue, the National Institutes of Health (NIH) created the All of Us Research Program, which focuses on creating a diverse and representative group of individuals in the United States. To achieve their goal, they identified the following ethnic and racial groups as “designated disparity groups” and referred to this group as underrepresented biomedical research (UBR) -Blacks/African Americans, American Indians/Alaska Natives, Asian Americans, Native Hawaiians and other Pacific Islanders, Hispanics/Latinos, and underserved rural populations [10, 11]. These groups have all been historically underserved, making research to account for demographic variability in clinical trials even more crucial.

Despite the movement being of utmost importance, there are no methods to predict the rate of UBR (rUBR). We address that gap by proposing a Bayesian method to predict and monitor overall accrual and rUBR. We applied this methodology to two completed trials and demonstrated accurate predictions of the rUBR at various timepoints within a trial. In the future, we will apply our methods to other inclusive research initiatives, such as All of Us, in the pursuit of giving a researcher the ability to make decisions from predictions of overall accrual and rUBR.

## Methods

### Model

The goal of our method is to provide simultaneous predictions of overall accrual and rUBR. We first review the overall accrual prediction model [7] before introducing the rUBR prediction. Suppose a researcher wants to monitor a trial to see if the trial sample size target will be met. Let $T$ be a fixed time from study initiation that the researcher sets for completing recruitment of a target sample size of *n* participants. Analyses can be used throughout to predict the probability that the target enrollments will be met by the end of the study. The overall accrual algorithm [7] define waiting time as the difference in dates of patients enrolling into the study [11]. Let ${t}_{1}$, ${t}_{2}$, …, ${t}_{m}$ represent the time a new participant is enrolled [7]. Assuming the study starts at time ${t}_{0}$, waiting times are defined as, ${w}_{i}={t}_{i}-{t}_{i-1}$. Taking the sum of the waiting times, $\sum_{i}^{m}{w}_{i=}$ ${t}_{m}$, where ${t}_{m}$ represents the time it took to accrue *m* participants into the clinical trial, these are the observable waiting times.

To account for the yet to be observed waiting times [7] to enroll $n-m$ participants, a model for the posterior predictive distribution of waiting times, ${W}_{m+1},{W}_{m+2},\dots,{W}_{n}$ is constructed. They assume the waiting times, ${w}_{i}|\theta$ are exponentially distributed given the mean, $\theta$ [7]. A conjugate prior for $\theta$ is set to an inverse gamma distribution - that is, $\theta\sim{IG}(k,V)$. These parameters are defined as $k$ = *nP* and $V$ = *TP*. The shape-scale parameters, $k$ and $V$, are functions of the confidence, $P$, in accruing $n$ participants where confidence $P$ is defined as how confident on a scale of 1–10 a researcher is in accruing $n$ participants within time *T*. The value of $P$ is then divided by 10 giving a number between 0 and 1 that describes a researcher’s confidence on the prior sample size. A value of $P=0$ would indicate a flat prior, meaning no confidence, while a value of $P=1$ would be a highly informative prior, meaning complete confidence of meeting the target sample size. This prior confidence is a constant prior used throughout the prediction. However, there is research for different types of confidence priors, such as accelerated or hedging priors [12]. Using the inverse gamma prior for $\theta$, they derive a conjugate inverse gamma posterior distribution. To find the yet to be observed waiting times, ${\theta}_{1}$ is randomly drawn from the posterior which is then used to randomly draw from the yet to be observed waiting times, ${W}_{m+1}\left({\theta}_{1}\right),{W}_{m+2}\left({\theta}_{1}\right),\dots,{W}_{n}\left({\theta}_{1}\right)$. This process is repeated for ${\theta}_{1,},{\theta}_{2},...,{\theta}_{s}$ where *s* is a large number. The total waiting time for the *j*^th^ draw in the trial of size *n* is then the sum of the observed waiting times and the posterior predictive waiting times, $$\begin{aligned}{L}_{j}\left(n\right)&={w}_{1}+{w}_{2},\dots+{w}_{m}+{W}_{m+1}\left({\theta}_{j}\right)\\&+{W}_{m+2}\left({\theta}_{j}\right)+\dots+{W}_{n}\left({\theta}_{j}\right),j=1,\dots,s\end{aligned}$$ [7]. The purpose of this method is to use waiting times to compute a predicted sample size. To compute the *j*^th^ posterior predictive sample size, ${n}_{j}^{p},$ where the superscript, $p$ is used to label a predicted value, partial sums of the total waiting times, ${L}_{j}\left(n\right)$ are calculated until the partial sums exceed *T*. The values ${n}_{j}^{p}$ are the largest values where the partial sums do not exceed *T*, and ${\{n}_{j}^{p},j=1,\dots,s\}$ gives an empirical distribution of the predictive sample size [7]. Throughout this paper, simulation is used to estimate the posterior distribution of parameters, as well as the associated posterior predictive distributions.    

Now, suppose a researcher wishes to also predict the rUBR throughout the study. Since being UBR can contain multiple categories, the rUBR can also consist of multiple categories, such as race, rurality, and ethnicity. In trials, these categories typically have separate rUBR goals of achieving a certain percentage within a total rUBR goal consisting of all categories. For example, a study could prespecify a total target of 60% rUBR and successfully meet this goal by enrolling 45% of the participants who are nonwhite, 10% who are rural, and 5% who are Hispanic. To introduce the rUBR predictions, let ${y}_{i}^{u}$= 1 if subject *i* is in the ${u}^{th}$category, and let ${y}_{i}^{u}$ = 0 if a subject is not in the ${u}^{th}$category, and ${S}_{m}^{u}=\sum_{i=1}^{m}{y}_{i}^{u}$. This follows a binomial distribution, where ${S}_{m}^{u}$ ~ binomial (*m*,${p}^{u}$) and ${p}^{u}$ is the probability that a study participant is UBR in the ${u}^{th}$ category. Here, we define the UBR categories as $u$ where $u⋲\{NW,H,R\}$ that includes $NW$ as non-white, $H$ as Hispanic and $R$ as rural. The motivation to treat each of these categories of UBR as separate binomial random variables has largely to do with the type of data used in these predictions. Many reports, such as DSMB reports and published manuscripts, only provide aggregated counts of non-white, Hispanic and rural throughout a trial. These aggregated counts give totals for each separate UBR category along with the entire total enrollment. Since data are presented in a separate aggregated form for each category, each of these categories are modeled as separate binomial random variables. For example, aggregated data at the first interim may report a count of 10 non-white, 10 Hispanic and 5 rural participants with an overall enrollment total of 50 enrolled. Aggregated counts such as these do not capture the amount of overlap between categories of UBR. Therefore, there is a possibility that our method double counts multiple groups. If a researcher has available unaggregated data where the overlap between UBR categories is known, then this information can be used in prediction. We explore the unaggregated analysis more in the supplementary section. For the purposes of this paper though, our goal is to provide a prediction method for aggregated data.

To get predictions of the rUBR, we start by deriving the posterior distribution of parameter ${p}^{u}$. We will assume a conjugate prior ${p}^{u}\sim{Beta}({\alpha}^{u},{\beta}^{u})$. The beta distribution captures the prior number of successes and failures through the ${\alpha}^{u}$ and ${\beta}^{u}$ parameters, respectively. We define the prior number of successes parameter as ${\alpha}^{u}$ = ${p}_{0}^{u}nP$, where ${p}_{0}^{u}$ is the prior probability for a subject being UBR. The same confidence parameter, *P*, from the overall accrual algorithm is used here. Recall, the value of *P* determines the confidence a researcher has in achieving a sample size of size, $n$. The prior expected rUBR, ${p}_{0}^{u}$, can be set by the investigator as a target rUBR. Similarly, we assume ${\beta}^{u}$ = $(1-{p}_{0}^{u})nP$ ,where the ${\beta}^{u}$ parameter models the prior number of failures. We will thus use a prior expected rate of non-UBR $(1-{p}_{0}^{u})$ to model the failures. Using the binomial model, we then construct likelihood of parameters $\alpha$ and $\beta$ given data to use with the beta prior, creating a posterior distribution of the parameter, ${p}_{j}^{u}$. Using conjugacy of the beta-binomial and observed data at time *t*_*m*_ – results in a posterior of

$${p}_{j}^{u}|{S}_{m}^{u}\sim{Beta}\;({\alpha}^{u}+{S}_{m}^{u},{\beta}^{u}+m-{S}_{m}^{u})$$

To find a future rate of UBR value, we achieve a posterior predictive distribution of a predicted rUBR value by averaging the sampling distribution over the posterior distribution of parameters. Since we have observed data up to $m$ participants thus far in the trial, our prediction of rUBR averages over the remaining ${n}_{j}^{p}-m$ sample size of rUBR where ${n}_{j}^{p}$ is the predicted overall accrual target derived earlier. Using simulation, the posterior predictive distribution of rUBR can then be modeled as,

$${S}_{{n}_{j}^{p}-m}^{u}|{p}_{j}^{u},{n}_{j}^{p}\sim{Binomial}\;({n}_{j}^{p}-m,{p}_{j}^{u}).$$

Since $${n}_{j}^{p}$$ is not a fixed value but instead a predictive accrual distribution, simulation is used to get the predictive distribution of rUBR. If instead a fixed $$n$$ was used, then distributional properties of the beta-binomial given the fixed sample size could be used to get a predictive distribution of rUBR. Summing the observed ($${S}_{m}^{u})$$ and predicted ($${S}_{{n}_{j}^{p}-m}^{u})$$rUBR values, the final predicted rUBR ($${S}_{{n}_{j}^{p}}^{u})$$ becomes,

$${S}_{{n}_{j}^{p}}^{u}={S}_{m}^{u}+{S}_{{n}_{j}^{p}-m}^{u}.$$.

Using non-white, Hispanic, or rural as variables that make up categorical UBR, a prediction model of UBR considers both the (${S}_{m}^{u}$) and (${S}_{{n}_{j}^{p}-m}^{u}$). To calculate the rUBR among all UBR categories we take the sum of proportions for each category. For non-white, we take the proportion of the observed non-white participants added with the predicted non-white participants over the entire predicted overall accrual. Likewise, this process is applied to Hispanic and rural the same way.

Combining the categories of non-white, Hispanic, and rural participants, the model to calculate rUBR is then:

$$rUBR_j^p=\frac{S_{n_j^p}^{NW}}{n_j^p}+\frac{S_{n_j^p}^H}{n_j^p}+\frac{S_{n_j^p}^R}{n_j^p}$$

By combining each of these categories we are providing an estimative rate for all UBR categories. At the end of the trial, the final ($$f$$) observed $${\mathrm{r}\mathrm{U}\mathrm{B}\mathrm{R}}_{f}$$ at the final sample size ($${n}_{f})$$ is:

$$rUB{R}_{f}=\frac{{n}_{f}^{NW}}{{n}_{f}}+\frac{{n}_{f}^{H}}{{n}_{f}}+\frac{{n}_{f}^{R}}{{n}_{f}}.$$

Where the variables, ${n}_{f}^{NW}$, ${n}_{f}^{H}$, and ${n}_{f}^{R}$ indicate the final total number of nonwhite (NW), Hispanic (H), and rural (R) participants, respectively. The variable for rural participants, ${n}_{f}^{R},$ is included for clarity as it is a crucial UBR category, although it will not be used in the two motivating examples. Thus, in our application of the method, we will focus on the prediction of non-white and Hispanic participants:

$${rUBR}_{j}^{p}=\frac{{S}_{{n}_{j}^{p}}^{NW}}{{n}_{j}^{p}}+\frac{{S}_{{n}_{j}^{p}}^{H}}{{n}_{j}^{p}}.$$

We demonstrate this methodology using only race and ethnicity. However, in *All of Us*, the UBR category may also include age, sexual and gender minorities, income, education, geography, disability, and health care access [13]. Through estimation, we provide a robust monitoring method for predicting overall accrual and the rUBR. Predicting these two simultaneously gives the researcher a more appropriate view of their enrollment needs. In this paper we acknowledge that we could simply provide predictions of each UBR subcategory separately. However, that does not provide a comprehensive prediction of UBR. The goal of a study is to enroll a target number of UBR participants where the rate of UBR is defined as per NIH as the combination of multiple underrepresented groups. By combining each category, we satisfy this goal. Further, we believe combining makes the prediction method more appealing to those wishing to use this prediction method in their study. Using data from completed real-world trials, we apply our method to two motivating examples that show how this prediction monitoring process could have been used in the trials. Through simulation, we illustrate how accurate the predictions are in meeting the actual overall accrual and *rUBR*_*f*_ targets. For each of these examples, we ran *N* = 10,000 iterations per prediction.

## Results

### Application of the ADORE trial data

**Fig. 1:**
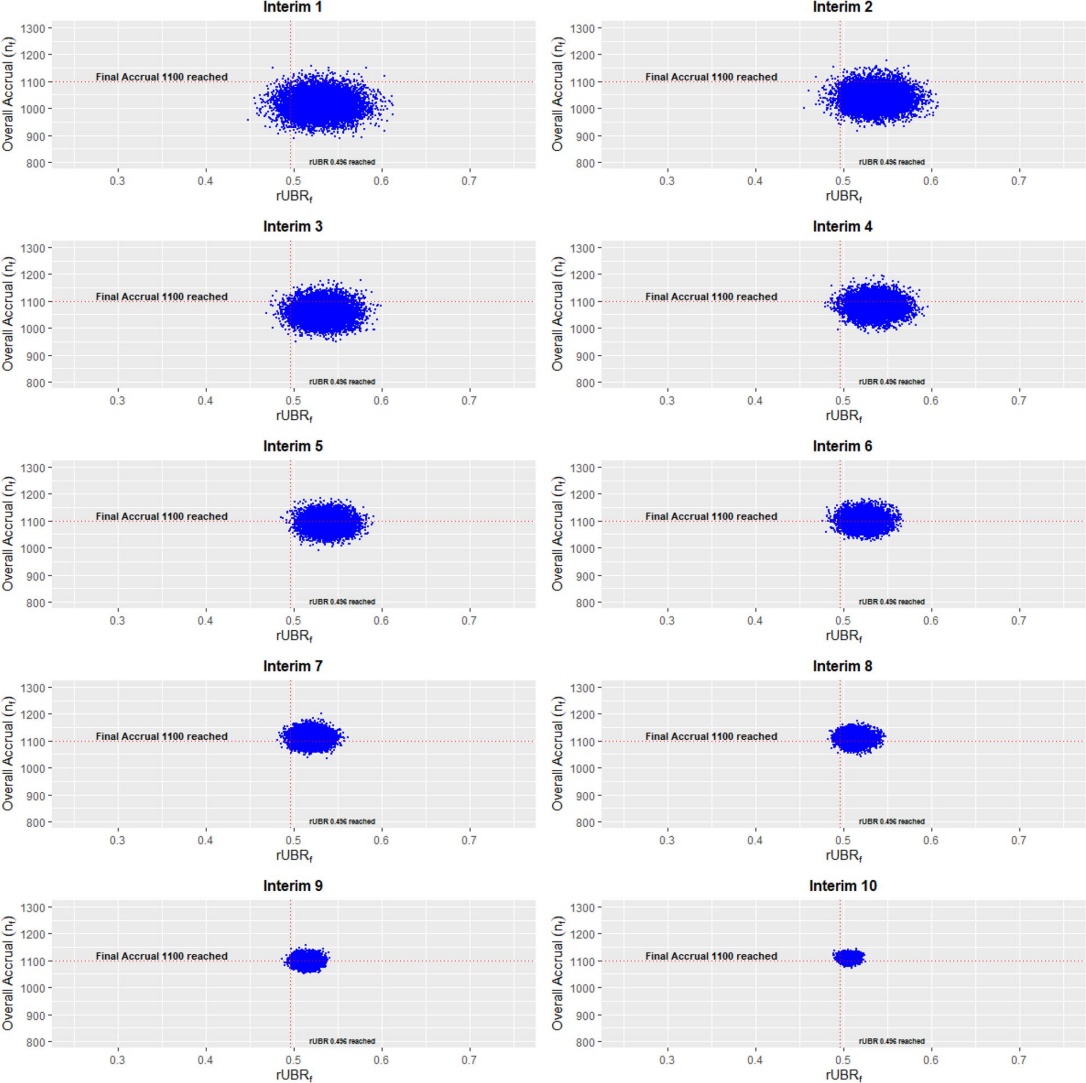
This figure shows predicted overall accrual and rUBR for ADORE trial data. In this trial, 10 interim analyses were done. The final overall accrual reached was 1100 participants (horizontal red dashed line), and the final rUBRf was 0.496 (vertical red dashed line). Ten thousand predicted values for overall accrual and rUBR are displayed

We apply our approach to the completed trial, An Assessment of DHA on Reducing Early preterm birth (ADORE) [14] (NCT02626299). ADORE was a multicenter, double-blind, randomized, adaptive superiority clinical trial designed to assess Docosahexaenoic acid (DHA) on reducing early preterm births (EPB) in women. The primary purpose of this trial was to determine if participants administered 1000 mg of DHA would have lower rates of EPB than those administered 200 mg of DHA. In this trial, investigators planned to recruit $n$ = 1355 participants by *T* = 48 months. Interim analyses were prespecified every 13 weeks after 300 new participants had been enrolled in the trial [14]. By the end of this trial, only ${n}_{f}$_*=*_ 1100 participants were enrolled over 45.2 months. Although the total number of participants was less than their initial target sample size, 1100 was sufficient for this trial. This trial collected demographic enrollment data, including race and ethnicity. During the monitoring phase, we first set prior confidence, *P*, which researchers typically choose from a range of 0.1–0.5. In our forecasts, we set a confidence level of $P$ = 0.3 of meeting the target accrual of $n$ = 1355. In this trial of 1100 final participants, 302 were non-white and 244 were Hispanic. Therefore, the final $rUB{R}_{f}=\frac{302}{1100}+\frac{244}{1100}=0.496$. Table 1 includes the predicted estimates for overall accrual and the rUBR with corresponding 95% credible intervals for each interim. In addition, from Fig. 1 the predictions are shown across each interim. The variation across the predicted overall accrual and rUBR decrease and approach the calculated true target values as the trial progresses.

**Table 1:** ADORE dataset predicted overall accrual estimates and rUBR estimates with corresponding 95% credible intervals per interim

Interim	Predicted Sample Size (95% CI)	Predicted rUBR (95% CI)
1	1014 (941, 1092)	0.53 (0.49, 0.57)
2	1037 (970, 1106)	0.54 (0.50, 0.58)
3	1058 (997, 1119)	0.53 (0.50, 0.57)
4	1079 (1024, 1134)	0.54 (0.51, 0.57)
5	1089 (1041, 1140)	0.54 (0.51, 0.57)
6	1099 (1056, 1143)	0.52 (0.50, 0.55)
7	1110 (1073, 1149)	0.52 (0.50, 0.54)
8	1106 (1075, 1139)	0.51 (0.50, 0.53)
9	1096 (1072, 1123)	0.51 (0.50, 0.53)
10	1107 (1090, 1125)	0.51 (0.50, 0.52)

**Fig. 2:**
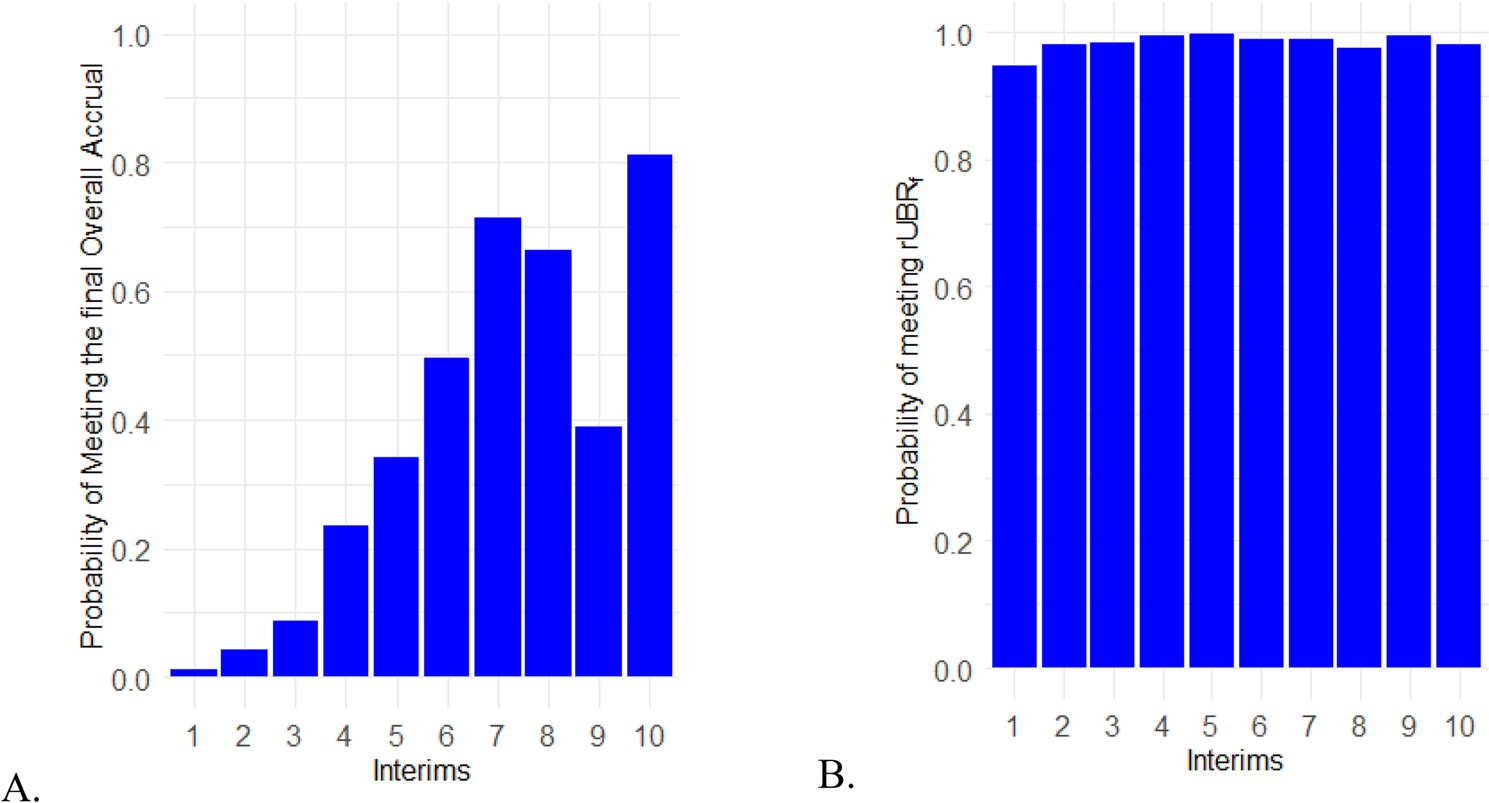
Probability of meeting the overall accrual and rUBR for each of the interims. **A** is the probability of meeting the final overall accrual, and **B** is the probability of meeting the *rUBR*_*f*_

We also calculated the probability to achieve the final overall accrual and *rUBR*_*f*_. For the final overall accrual of 1100, the calculation goes like this: for the *j*^th^ simulation, if the value is greater than 1100 it would indicate that the final accrual was met. If a prediction for the *j*^th^ simulation is less than 1100 then it did not meet the final accrual. This is similarly done with a *rUBR*_*f*_ of 0.496. Taking the proportion of simulations above for both final overall accrual and the final *rUBR*_*f*_ gives us the probability of meeting overall accrual and the *rUBR*_*f*_, respectively (Fig. 2). The probability of meeting the final overall accrual of 1100 increased for each interim, except during the 8th and 9th interims (Fig. 2A.). For every interim, the probability of meeting the final *rUBR*_*f*_ was always greater than 90% as shown in (Fig. 2B.). This demonstration of our method on the ADORE trial data shows that predictions of overall accrual and rUBR can be used to assess the probability of reaching final overall accrual and *rUBR*_*f.*_

## Application to Quit2Live data

**Fig. 3:**
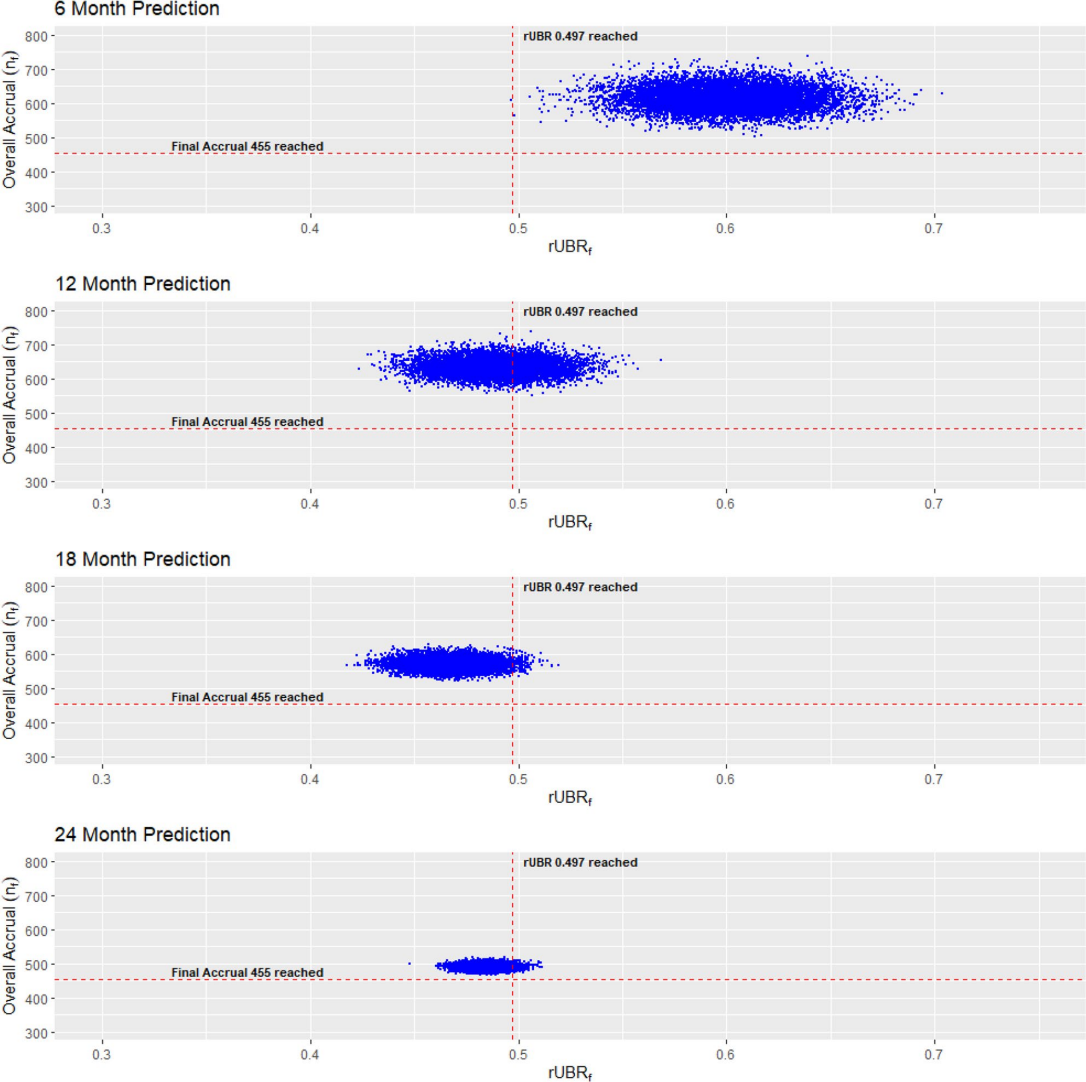
Quit2Live predictions for overall accrual and rUBR calculated at four different timepoints throughout the trial. For this trial, the true overall accrual was 455 participants (horizontal red dash line), and the final *rUBR*_*f*_ was 0.497 (vertical red dashed line)

**Fig. 4:**
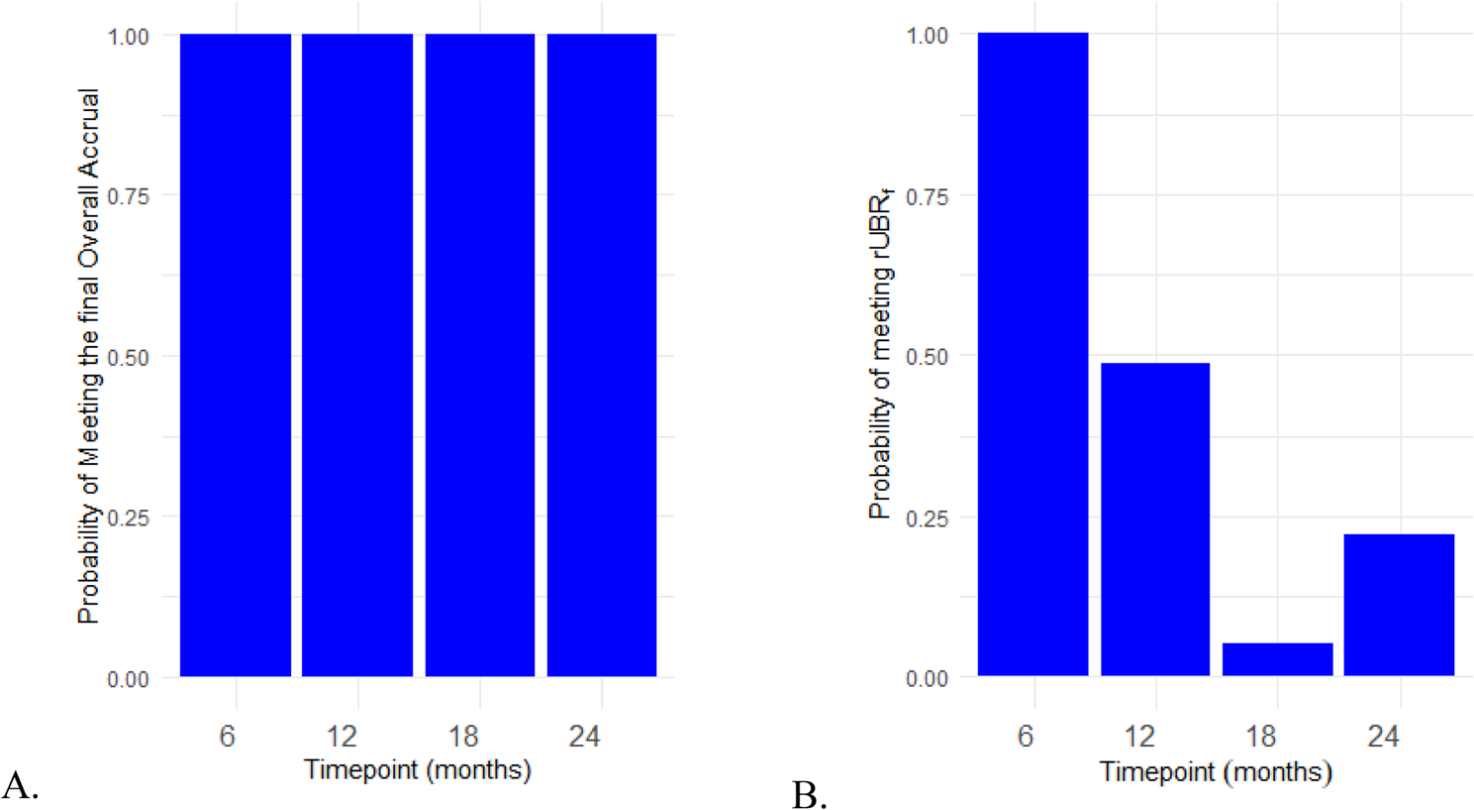
Probability of meeting the truth at each timepoint for overall accrual and rUBR. **A** is the probability of meeting the overall accrual per timepoint and **B** is the probability of meeting the *rUBR*_*f*_

We further demonstrated our method by applying it to Quit2Live, a five-year prospective cohort intervention trial that examined disparities in smoking cessation between African Americans and White adult smokers [15] (NCT01836276). Participants were enrolled from February 2013 until May 2015. The eligibility criteria included participants who were at least 18 years of age and who smoked 3–20 cigarettes per day [15]. The planned enrollment time to achieve an overall accrual target of $n=$455 was $T=$2.5 years (30 months) with a 6-month follow-up period. In this trial, unlike the ADORE trial, there were no Hispanic participants, and there were no interim analyses. Of the participants, 226 were African American and 229 were white. Although interim analyses weren’t done in the trial, we can still make predictions of overall accrual and the rUBR using our method at any point throughout the trial. Therefore, we analyzed this dataset every 6 months within the 30-month trial duration. From the dataset, there were 226 non-white participants enrolled and zero Hispanic participants, and thus the actual $rUB{R}_{f}=\frac{226}{455}+\frac{0}{445}=0.497$. Like the ADORE example, we create predictions for of the overall accrual and rUBR simultaneously (Fig. 3) for the Quit2Live dataset. In the Quit2Live trial, the variation of our predicted values decreased as the trial continued up to a point where all the predicted values are close to the actual overall accrual and $rUB{R}_{f}$. Intuitively, this makes sense, as toward the end of the trial, the observed data is mostly driving the prediction. The change in variation across predicted values per time point is shown in Table 2 with credible intervals. We also find that the probability of meeting the overall accrual was always 100% (Fig. 4A.). For the predicted rUBR we find that early in the trial, the probability of meeting the actual $rUB{R}_{f}$ was close to 100% but it later decreased to 5% before increasing again close to 25%. In this example, our prediction method, if applied during the trial, could have assisted the monitoring process by showing how close the researchers were to achieving their overall accrual and rUBR targets.

**Table 2:** Quit2Live dataset predicted overall accrual estimates and rUBR estimates with corresponding 95% credible intervals for the four different timepoints

Timepoint (months)	Predicted Sample Size (95% CI)	Predicted rUBR (95% CI)
6	613 (552, 677)	0.60 (0.55, 0.65)
12	632 (588, 677)	0.49 (0.45, 0.53)
18	569 (541, 600)	0.47 (0.44, 0.49)
24	489 (475, 503)	0.48 (0.47, 0.50)

## Discussion

By applying our method to two different clinical trials, we have demonstrated the accuracy of our predictions and their usefulness as a monitoring process. From the ADORE trial, our forecasts of overall accrual and rUBR per interim show the predicted values approaching the actual overall accrual and *rUBR*_*f*_ by the end of the trial. Throughout the ADORE trial, we show a greater than 90% probability of meeting the target UBR at any point during the trial. Our findings from the Quit2Live trial are similar. At 18 months, the probability of meeting the actual *rUBR*_*f*_ is small, approximately 5%. This information is crucial to researchers as it enables them to evaluate the feasibility of continuing the trial, given the resources they have. Recruitment of UBR participants can be challenging, and a probability of 5% at 18 months may not be sufficient to prompt active outreach efforts for UBR enrollment during the remainder of the trial. Unfortunately for the Quit2Live researchers, they did not have these predictions. However, they did observe a low number of UBR participants at 18 months and decided to try and enroll more UBR participants. This explains the 20% increase in UBR enrollment between 18 and 24 months. In the future, our method can be used to avoid this issue, allowing researchers to accurately predict the number of UBR participants required to enroll to meet their target before the trial is nearing its completion.

In our assessment of predictions for overall accrual and rUBR we use the probability of meeting the *actual* overall accrual and *rUBR*_*f*_. This is a consequence of using completed data for the two trials used in this paper. If instead these predictions were done as part of a trial monitoring process at some point during the trial, then a target set by the researcher for overall accrual and target final rUBR should be used.

A limitation of using the actual *rUBR*_*f*_ in our performance assessments could be that the population reflects a low percentage of those considered UBR where some communities could have UBR percentages around 10–20%. In these instances, if a researcher had a goal of 50% UBR participants for example, this may be difficult to achieve if at enrollment limited effort is dedicated to recruiting UBR participants. In such cases, it is possible that our method of prediction and monitoring could only achieve percentages close to the true population rUBR since our method uses observed data to drive the prediction.

One of the assumptions of our prediction modeling was setting a prior confidence, $P$. In our prediction we set a prior confidence of $P=0.3$, although this number can be chosen depending on study needs. $P$ usually ranges from 0.1 to 0.5. A researcher should consider this confidence parameter in relation to how confident they are in achieving the sample size in their study. We also set the prior probability of being UBR for each category of UBR, along with a prior assumption, on the overall accrual before trial data has been collected. Specifications of these priors are trial-oriented and should be decided carefully.

A limitation of using aggregated data is that several UBR categories can have overlap of one or more categories, and this overlap cannot be accounted for using our method. For example, in some cases, an individual considered UBR could be both non-white and Hispanic, but our method would count them once for each of these categories. In the case that there exists extensive overlap between categories this would create an overestimated rUBR value. In the case that there is little overlap the predictions would not be affected significantly. This consequence of using aggregated data should be considered when using our prediction method. In the supplementary section though, we argue that the beta-binomial framework used in our prediction algorithm, can be applied very similarly on unaggregated data.

## Conclusion

Using two existing, real-world datasets, we have shown that our method of prediction and monitoring can be used throughout a trial to predict a trial’s overall accrual and rUBR at any point in the trial or study.

## Supplementary Information

Supplementary Material 1.

## Data Availability

Contact corresponding author for data.

## References

[1] Carlisle B, Kimmelmen J, Ramsay T, MacKinnon N. Unsuccessful Trial Accrual and Human Subjects Protections: An Empirical Analysis of Recently Closed Trials. Clin Trails. 2015;12(1):77–83. 10.1177/1740774514558307. 10.1177/1740774514558307 PMC4516407 25475878

[2] Cheng S, Dietrich M, Dilts D. Predicting Accrual Achievement: Monitoring Accrual Milestones of NCI-CTEP–Sponsored Clinical Trials. Clin Cancer Res. 2011;17(7):1947–55. 10.1158/1078-0432.CCR-10-1730. 21447723 10.1158/1078-0432.CCR-10-1730 PMC3074352

[3] Kitterman D, Cheng S, Dilts D, Orwall E. The Prevalence and Economic Impact of Low-Enrolling Clinical Studies at an Academic Medical Center. Acad Res. 2011;86(11):1360–6. 10.1097/ACM.0b013e3182306440. 10.1097/ACM.0b013e3182306440 PMC3203249 21952064

[4] Senn S. Some controversies in planning and analysing multi-centre trials. Stat Med. 1998;17:1753–6. 10.1002/(sici)1097-0258(19980815/30)17:15/16<1753::aid-sim977>3.0.co;2-x 9749445

[5] Anisimov VV, Fedorov VV. Modelling, prediction and adaptive adjustment of recruitment in multicentre trials. Stat Med. 2007;26:4958–75. 10.1002/sim.2956. 17639505 10.1002/sim.2956

[6] Abbas I, Rovira J, Casanovas J. Clinical trial optimization: Monte Carlo simulation Markov model for planning clinical trials recruitment. Contemp Clin Trials. 2007;28(3):220–31. 10.1016/j.cct.2006.08.002. 16979387 10.1016/j.cct.2006.08.002

[7] Gajewski BJ, Simon SD, Carlson SE. Predicting accrual in clinical trials with Bayesian posterior predictive distributions. Stat Med. 2008;27(13):2328–40. 10.1002/sim.3128. 17979152 10.1002/sim.3128

[8] All of Us Research Program Investigators. The All of Us Research Program. N Engl J Med. 2019;381:668–76. 10.1056/NEJMsr1809937. 31412182 10.1056/NEJMsr1809937 PMC8291101

[9] Bianchi DW, Brennan PF, Chiang MF, et al. The All of Us Research Program is an opportunity to enhance the diversity of US biomedical Research. Nat Med. 2024;30:330–3. 10.1038/s41591-023-02744-3. 38374344 10.1038/s41591-023-02744-3 PMC11835384

[10] National Institute on Minority Health and Health Disparities. Minority Health and Health Disparities Definitions. National Institute on Minority Health and Health Disparities. https://www.nimhd.nih.gov/resources/understanding-health-disparities/minority-health-and-health-disparities-definitions.html. Accessed 18 Nov 2024.

[11] Mapes BM, Foster CS, Kusnoor SV, et al. All of Us Research Program. Diversity and inclusion for the All of Us research program: A scoping review. PLoS ONE. 2020;15(7):e0234962. 10.1371/journal.pone.0234962. 32609747 10.1371/journal.pone.0234962 PMC7329113

[12] Jiang Y, Simon S, Mayo MS, Gajewski BJ. Modeling and validating Bayesian accrual models on clinical data and simulations using adaptive priors. Stat Med. 2015;34(4):613–29. 10.1002/sim.6359. 25376910 10.1002/sim.6359 PMC4314351

[13] How does All of Us assess diversity? What communities does All of Us consider. underrepresented in biomedical research. National Institute of Health. How does All of Us assess diversity? What communities does All of Us consider underrepresented in biomedical research (UBR)? – All of Us Research Hub. Accessed 18 Nov 2024.

[14] Carlson SE, Gajewski BJ, Valentine CJ, et al. Higher dose docosahexaenoic acid supplementation during pregnancy reduces early preterm birth: a randomised, double-blind, adaptive-design superiority trial. EClinicalMedicine. 2021;17(36):100905. 10.1016/j.eclinm.2021.100905. 10.1016/j.eclinm.2021.100905 PMC8257993 34308309

[15] Nollen NL, Cox LS, Yu Q, et al. A clinical trial to examine disparities in quitting between African-American and White adult smokers: Design, accrual, and baseline characteristics. Contemp Clin Trials. 2011;32(6):769–77. 10.1016/j.cct.2015.12.001. 10.1016/j.cct.2015.12.001 PMC4818177 26667382

