# Supplementary material for: Prediction and monitoring of accrual and rate of underrepresented biomedical research group using bayesian methods
Source: BMC Med Res Methodol. 2026 Mar 19;26:96. doi: 10.1186/s12874-026-02822-3 (PMC13122904; doi:10.1186/s12874-026-02822-3)
Supplement: Supplementary file 1 — Supplementary Material 1. [file 12874_2026_2822_MOESM1_ESM.docx]

**Supplementary Material**

**Unaggregated Analysis of rUBR**

Suppose we have unaggregated data showing participant level overlap between the UBR categories that include, race, ethnicity and rurality. For race, an individual is classified as being white (W) or non-white (NW). For ethnicity, an individual is classified as being Hispanic (H) or non-Hispanic (NH). For rurality an individual is classified as being rural (R) or non-rural (NR).

If we treat each of these combinations as non-mutually exclusive meaning, there is overlap of categories then there is a possible $2^{3}$ different combinations. In the following table we provide the possible combinations along with corresponding probability of being in each combination, $p_{1},p_{2}, \ldots, p_{8}$ with $\sum_{i=1}^{8} p_{i}$ = 1:

| **Race** | **Ethnicity** | **Rurality** | **Probability** |
| --- | --- | --- | --- |
| NW | H | R | $p_{1}$ |
| NW  W  W  NW  NW | NH  H  NH  H  NH | R  R  R  NR  NR | $p_{2}$  $p_{3}$  $p_{4}$  $p_{5}$  $p_{6}$ |
| W | H | NR | $p_{7}$ |
| W | NH | NR | $p_{8}$ |

From the table we can compute the marginal probabilities of each the UBR categories:

$P_{R}$ = $p_{1}$ + $p_{2}$ + $p_{3}$ + $p_{4}$

$P_{NW}$ = $p_{1}$ + $p_{2}$ + $p_{5}$ + $p_{6}$

$P_{H}$ = $p_{1}$ + $p_{3}$ + $p_{5}$ + $p_{7}$

*Note: in this manuscript the final study results are reported as* $P_{R}+P_{NM}+P_{H}$ *which may be >1. This has to be done because we have aggregated data (i.e. only the marginals).*

Next, we can find the intersection among pairs and all three UBR categories:

$P_{R, NW}= P(R \cap NW)$ = $p_{1}$ + $p_{2}$

$P_{NW, H}= P\left( NW \cap H \right)$ = $p_{1}$ + $p_{5}$

$P_{R, H}= P(R \cap H)$ = $p_{1}$ + $p_{3}$

$P_{R, H, NW}= P(R \cap H\cap NW)$ = $p_{1}$

To account for the overlap of multiple categories when calculating the probability of being UBR we would find the union of all three groups. We define the probability of a participant being UBR ($p_{UBR}$):

$p_{UBR} =$ $P(R \cup H\cup NW)$ = $P_{R}$ + $P_{NW}$ + $P_{H}$ - $P_{R, NW}$ - $P_{NW, H}$ - $P_{R, H}$ + $P_{R, H, NW}$

= $p_{1}$ + $p_{2}$ + $p_{3}$ + $p_{4}$ + $p_{1}$ + $p_{2}$ + $p_{5}$ + $p_{6}$ + $p_{1}$ + $p_{3}$ + $p_{5}$ + $p_{7}$ - $p_{1}$ - $p_{2}$ - $p_{1}$ - $p_{5}$ - $p_{1}$ - $p_{3}+$ $p_{1}$

= $p_{1}$ + $p_{2}$ + $p_{3}$ + $p_{4}$ + $p_{3}$ + $p_{4}$ + $p_{7}$

= $\sum_{i=1}^{7} p_{i}$

= 1 - $p_{8}$

From the calculation, the $p_{UBR}$would be the sum of marginal probabilities of each of the categories that contain a UBR category minus the category that does not include any UBR category, $p_{8}$. With unaggregated data, each of these marginal probabilities are known. A beta-binomial model can be applied in the same manner as shown with aggregated data. In the main text of the manuscript, we model aggregated data for each individual category of UBR and assume a binomial distribution for a participant being UBR or not UBR for each of the categories of UBR. Similarly, here we can group all participants with probability, $\sum_{i=1}^{7} p_{i}$ as being UBR and $p_{8}$ as not UBR. The algorithm used in the manuscript would follow the same logic for calculating rUBR.

**Aggregated Analysis R Code Example**

R Code is provided that shows the methodology using R code. The ADORE trial data is used as a dataset example in the code.

# Packages Needed

install.packages("accrual")

install.packages("tidyverse")

install.packages("ggpubr")

install.packages("ggplot2")

library(tidyverse)

library(ggpubr)

library(accrual)

library(ggplot2)

# Function for each interim and UBR model. Input is sample size(n),End time(T), #Confidence(P),

# observed participants (m), time when m participants accrued (Tp),observed nonwhite #(nonwhite),

# observed Hispanic (Hispanic), target nonwhite percentage (p0),target Hispanic percentage.

interims <- function(n,T,P,m,tm,Tp,nonwhite,Hispanic,p0,p1) {

# Function for each interim used to get Predicted sample size.

Npred <- accrual.n.inform(n=n, T=T, P=P, m=m, tm=tm, Tp=Tp)[[2]]

# Length of Npred used in simulation

end <- length(Npred)

# Initialize the values

p <- ph <- y <- yh <- UBR <- numeric(end)

# Beta prior for non-white, using target sample size, n

alpha1=p0*n*P

beta1=(1-p0)*n*P

# Beta prior for Hispanic, using target sample size, n

alpha2=p1*n*P

beta2=(1-p1)*n*P

# Posterior Distribution of non-white

p <- rbeta(10000,alpha1 + nonwhite, m + beta1 - nonwhite)

# Posterior Predictive Distribution

y = rbinom(10000, Npred-m, p)

# Posterior Distribution of Hispanic

ph <- rbeta(10000,alpha2 + Hispanic, m + beta2 - Hispanic)

# Posterior Predictive Distribution

yh = rbinom(10000, Npred-m, ph)

# UBR for Nonwhite and Hispanic

UBR = (y+nonwhite)/Npred + (yh+Hispanic)/Npred

dat <- data.frame(UBR, Npred)

return(dat)

}

# Example, Application of Data

interim1 <- interims(1355,48,0.3,301,15.3,45.2,98,68,0.45,0.05)

interim2 <- interims(1355,48,0.3,386,18.3,45.2,112,102,0.45,0.05)

interim3 <- interims(1355,48,0.3,473,21.4,45.2,142,116,0.45,0.05)

interim4 <- interims(1355,48,0.3,558,24.3,45.2,167,137,0.45,0.05)

interim5 <- interims(1355,48,0.3,640,27.3,45.2,192,157,0.45,0.05)

interim6 <- interims(1355,48,0.3,723,30.3,45.2,215,166,0.45,0.05)

interim7 <- interims(1355,48,0.3,805,33.2,45.2,238,182,0.45,0.05)

interim8 <- interims(1355,48,0.3,878,36.2,45.2,257,195,0.45,0.05)

interim9 <- interims(1355,48,0.3,945,39.2,45.2,277,210,0.45,0.05)

interim10 <- interims(1355,48,0.3,1031,42.2,45.2,294,228,0.45,0.05)

# Function to generate a plot for all interim datasets

plot_function <- function(interim_data, title) {

ggplot() +

labs(y = "Predicted Accrual (N)", x = "UBR") +

geom_point(aes(x = UBR, y = Npred, colour = title), data = interim_data, color = "blue") +

geom_hline(yintercept = 1100, linetype = 'dotted', col = 'red') +

annotate("text", x = 0.35, y = 1100, size = unit(3, "pt"), label = "Sample Size 1100 reached", vjust = -0.25) +

geom_vline(xintercept = 0.496, linetype = 'dotted', col = 'red') +

annotate("text", x = 0.59, y = 805, size = unit(2, "pt"), label = "UBR 0.496 reached", hjust = -0.1) +

xlim(0.25, 0.75) + ylim(800, 1300) +

labs(colour = "Interims") + ggtitle(title)

}

# Store interim objects in a list

interim_data <- list(interim1, interim2, interim3, interim4, interim5,

interim6, interim7, interim8, interim9, interim10)

# Generate titles for the plots

titles <- paste("Interim", 1:10)

# Create the plots

plots <- lapply(seq_along(interim_data), function(i) plot_function(interim_data[[i]], titles[i]))

# Arrange All the Plots in a Grid

figure <- ggarrange(plotlist = plots, ncol = 2, nrow = 5)

# View the Figure

print(figure)
